# Efficacy of dietary supplementary probiotics as substitutes for antibiotic growth promoters during the starter period on growth performances, carcass traits, and immune organs of male layer chicken

**DOI:** 10.14202/vetworld.2022.324-330

**Published:** 2022-02-14

**Authors:** B. Agustono, W. P. Lokapirnasari, M. N. Yunita, R. N. Kinanti, A. E. Cesa, S. Windria

**Affiliations:** 1 Department of Veterinary Science, Division of Animal Husbandry, Faculty of Veterinary Medicine, Universitas Airlangga, Surabaya, Indonesia; 2 Department of Veterinary Science, Division of Pathology Veteriner, Faculty of Veterinary Medicine, Universitas Airlangga, Surabaya, Indonesia; 3 Faculty of Veterinary Medicine, Universitas Airlangga, Surabaya, Indonesia; 4 Department of Biomedical Sciences, Division of Microbiology, Veterinary Medicine Study Program, Faculty of Medicine, Universitas Padjadjaran, Bandung, Indonesia

**Keywords:** feed supplementation, growth performance, ISA brown layer chicken, probiotic, starter period

## Abstract

**Background and Aim:**

With the increased concerns about global protein supply, chicken meat, especially from male layer chicken, constitutes an alternative in terms of quality and carcass traits. Probiotics have been proposed for replacing antibiotic growth promoters (AGPs), which have been prohibited as poultry supplement feeds. The present study aimed to determine the efficacy of dietary supplementary probiotics during the starter period on growth performances, carcass traits, and immune organs of male layer chicken.

**Materials and Methods:**

In this study, one hundred and eighty 1-day-old male chicks from the strain ISA brown were used. They were divided into six groups according to the feed: 100% basal feed (T0), basal feed+2.5 g AGP/kg feed (T1), basal feed+probiotics 1 mL/kg feed (T2), basal feed+probiotics 3 mL/kg feed (T3), basal feed+probiotics 4 mL/kg feed (T4), and basal feed+probiotics 5 mL/kg feed (T5). Probiotics (*Lactobacillus acidophilus*, *Lactobacillus plantarum*, and *Bifidobacterium* spp.) were given at a concentration of 1.2×10^9^ colony-forming unit/mL. Virginiamycin was used as AGP. ISA brown layer chicken was treated for 21 days. Growth performances (body weight, feed consumption, and feed conversion ratio [FCR]), carcass traits (weight at slaughter, weight of the carcass, breast muscles, liver, lungs, kidneys, and heart), immune organs (spleen, thymus, and bursa of Fabricius), and non-edible organs (head, legs, and wings) were analyzed.

**Results:**

Probiotic supplementation at 4 and 5 mL/kg feed (T4 and T5) during the starter phase improved the body weight, FCR, and feed consumption. The weight at slaughter, weight of the carcass, breast muscles, and liver from the T4 and T5 groups were significantly greater than those in the other treatment groups. In addition, the weight of the heart, lungs, and kidneys was increased in the T1, T2, T3, T4, and T5 groups compared with that measured in the T0 group. Furthermore, there were significant differences regarding the immune organs between the T0 and the other treatment groups. The weight of the head, legs, and wings was also greater in the probiotic and AGP supplementation groups (T1, T2, T3, T4, and T5) than that in the basal feed group (T0).

**Conclusion:**

Probiotic (*L. acidophilus*, *L. plantarum*, and *Bifidobacterium* spp.) supplementation at 4 and 5 mL/kg feed during the starter period can be used to improve the growth, carcass traits, and weight of immune organs in male layer chicken.

## Introduction

Chicken meat is one of the preferred animal protein sources in the Indonesian region [1]. Indeed, it is very popular and widely consumed by local people and is currently obtained from broiler and native chicken. Medium male layer chicken is the meat-producing chicken of choice for the general public because the meat texture is similar to that of native chickens [2]. There are several advantages in using medium male layer chicken beside the meat quality, including the ease to market them, the cheaper cost of day-old chicks (DOCs), and the lower fat content compared with broilers [3,4]. DOCs are chicks in the early post-hatching period, which is considered a critical period for chicks [5]. The gastrointestinal tract of postnatal monogastric animals usually has an immature immune system, an unstable microbiota, and suboptimal endogenous enzyme secretion [6,7]. Farmers often use antibiotic growth promoters (AGPs) to improve the immune system in the starter phase. Administration of AGPs during the maintenance period of laying hens is also beneficial for the balance of the microbiota ecosystem and improves the digestibility of nutrients during the starter phase [8].

However, AGPs have long-term negative effects besides their beneficial effects on carcass quality and the immune system in laying hens [9]. Because of the detrimental effects of AGP administration (regarding both food safety and environmental impacts/residues) to chickens, the Indonesian government officially banned the use of AGPs as additives to animal feed in 2018 (Prohibition of the use of AGP in article 16 of the Regulation of the Minister of Agriculture Number 14 of 2017 concerning the Classification of Veterinary Drugs). The potential absorption of antibiotics in livestock products, including chicken meat, led to the prohibition of antibiotics as feed additives [10]. Using AGPs, low concentrations of antibiotics are ingested by consumers, potentially increasing chemical residues and bacterial resistance and causing allergic responses [11,12]. AGPs might be replaced with probiotics, and potential commercial applications of probiotic feed supplementation to enhance the growth performances of chicken have been shown [13].

Probiotics as non-antibiotics are used as alternative feed additives to improve digestion and absorption of nutrients in the intestines by supplying digestive enzymes, reducing the pH, and increasing enzymatic activities in the digestive tract of chickens [14-16]. Probiotic supplementations contain beneficial microbiota, such as *Lactobacillus* spp. Furthermore, *Bifidobacterium* spp. has a positive impact on the gastrointestinal microbiota population. Probiotics increase the activity of digestive enzymes and consequently improve food absorption. Chickens can utilize Well-absorbed food for tissue growth and weight gain [17,18]. Probiotics (*Lactobacillus acidophilus* and *Bifidobacterium* spp.) have been shown to reduce the feed conversion ratios (FCRs) and affect the consumption of chicken proteins. Various doses of probiotics have been tested, but the exact dosage setting remains to be determined [19].

This study aimed to determine the efficacy of dietary supplementary probiotics as substitutes for AGPs during the starter period on growth performance, carcass traits, and immune organs in male layer chicken.

## Materials and Methods

### Ethical approval

The study was approved by the Ethical Committee of Faculty of Veterinary Medicine, Universitas Airlangga, Indonesia, with number 518/HRECC.FODM/IX/2021.

### Study period and location

This study was conducted for two months (August and September 2021). The DOCs were reared in the breeding farm located at Faculty Veterinary Medicine, Universitas Airlangga. Proximate analysis of the feed was conducted at Laboratory of Animal Nutrition, Faculty of Veterinary Medicine, Universitas Airlangga. Variables examination was performed at the Laboratory of Animal Production, PSDKU Banyuwangi, Universitas Airlangga.

### Experimental design

This study followed a completely randomized design. A total of 180 ISA brown males aged 1 day in the starter phase were divided into six treatment groups. Each treatment group consisted of three replication subgroups of 10 ISA brown males. Animals were housed in individual cages for layer chicken and fed with the treatment twice a day, in the morning and evening. Drinking water and feed were provided *ad libitum*.

The feed used in this study was complete feed. The nutritional content for the starter period is listed in Table-1 [20]. In the control group, the feed was not supplemented with AGP or probiotics, (T1) basal feed+2.5 g AGP/kg feed namely, virginiamycin, was added in 1 kg of feed and the mixture was prepared. (T2) basal feed+probiotics 1 mL/kg feed, (T3) basal feed+probiotics 3 mL/kg feed, (T4) basal feed+probiotics 4 mL/kg feed, and (T5) basal feed+probiotics 5 mL/kg feed. A solution containing 1.2×10^9^ colony-forming unit/mL of the probiotics *L. acidophilu*s, *Lactobacillus plantaru*m, and *Bifidobacterium* spp. was sprayed on the ISA brown stud feed using a spray and then air-dried for 5-10 min. Each treatment lasted for 21 days.

**Table 1:** Ingredients and calculated analysis of basal diet (gram in 1 kg basal diet) [20].

Ingredient (g)	Starter
Corn	482.61
Soybean meal	302.45
Alfalfa meal	61.38
Poultry by-product meal	50
Poultry fat	70.62
Dicalcium phosphate	14.75
Limestone	9.72
Salt	3.03
DL-methionine	2.19
Vitamin-mineral premix	2.50
Coban	0.75
Total	1000
Calculated analysis (%)	
Dry matter	87
Ash	7
Extract ether	5
Crude fiber	5
ME (kcal/kg)	3.200
Crude protein	23
Crude protein	23.2
Calcium	1.0
Available phosphorus	0.5
Methionine+cysteine	0.93
Lysine	1.23
Threonine	0.97

ME=Metabolizable energy

### Sampling and measurements

The feed efficacy was calculated using the chicken body weight and feed consumption following the formulas below. It was evaluated on days 1, 14, and 21 at 7 a.m.

Feed consumption (g)=Amount of feed given (g)–Amount of unconsumed feed;

FCR=Feed consumption/Body weight;

Feed efficiency=(Body weight gain/Feed consumption)×100.

On 21^st^ day, the male layer chicken was sacrificed. The organs were collected to determine the carcass traits (weight of the animals at slaughter and weight of the carcass, breast muscles, liver, lungs, kidneys, and heart) and the weight of immune organs (spleen, thymus, and bursa of Fabricius) and non-edible organs (weight of the head, legs, and wings).

### Statistical analysis

The data collected during the study were analyzed using Statistical Package for the Social Sciences version 26.0 software (IBM Corp., NY, USA) to analyze the variance with p<0.05 as the significance threshold. If there was a significant effect, the differences among groups were assessed with Duncan’s multiple distance test (5%).

## Results

### Growth performances

**Figure-1:**
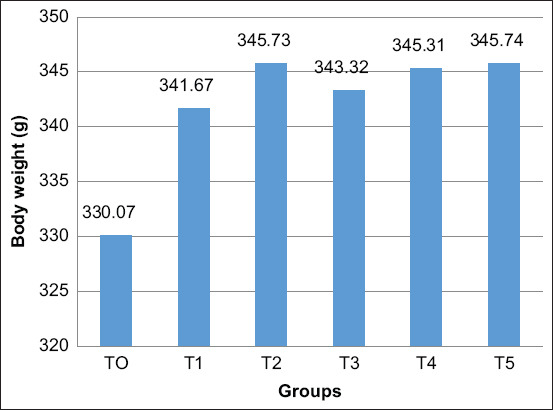
Efficacy of dietary supplementary probiotics on male layer chicken body weight during starter period. Values are expressed in mean±standard error. One-way analysis of variance was carried out followed by Duncan’s comparison test. ^a,b,c^Different superscripts indicate significant differences (p<0.05).

The growth performances of male layer chicken were assessed with body weight, FCR, and feed consumption. The body weight of animals from the T1 group was significantly increased than that from the T0 group (fed with 100% basal feed) (Figure-1). Furthermore, probiotic supplementation (*L. acidophilus*, *L. plantarum*, and *Bifidobacterium* spp.) at 1, 4, and 5 mL/kg feed (T2, T4, and T5 groups, respectively) induced an increase in the body weight of male layer chicken compared with that of the T1 group receiving AGP (virginiamycin) supplementation (Table-2 and Figure-1).

**Table 2:** Growth performance of male layer chicken fed different experimental diets during the starter period.

Variables	T0	T1	T2	T3	T4	T5
Initial weight (g)	42.560±0.105^a^	42.547±0.112^a^	42.573±0.109^a^	42.560±0.129^a^	42.560±0.118^a^	42.547±0.124^a^
Body weight (g)	330.07±6.15^a^	341.67±2.44^b^	345.73±4.94^c^	343.32±3.67^b,c^	345.31±2.37^c^	345.74±3.48^c^
Feed consumption (g)	934.7±3.5^a^	1015.8±3.8^b^	1018.7±3.8^b,c^	1011.7±5.5^b,c^	1018.2±3.8^c^	1014.9±4.3^c^
Feed conversion ratio (kg/kg gain)	3.040±0.04^a^	2.957±0.03^b^	2.964±0.03^b^	2.946±0.02^b^	2.890±0.03^c^	2.877±0.04^c^

^a,b,c^ Different superscripts in the same raw show significant difference (p<0.05). (T0) 100% basal feed, (T1) basal feed+2.5 g AGP/kg feed, (T2) basal feed+probiotic 1 mL/kg feed, (T3) basal feed+probiotics 3 mL/kg feed, (T4) basal feed+probiotic 4 mL/kg feed, and (T5) basal feed+probiotic 5 mL/kg feed. AGP=Antibiotic growth promoter

**Figure-2:**
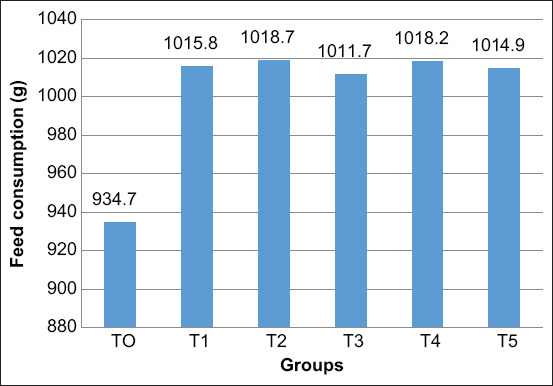
Efficacy of dietary supplementary probiotics on feed consumption of male layer chicken during the starter period. Values are expressed in mean±standard error. One-way analysis of variance was carried out, followed by Duncan’s comparison test. ^a,b,c^Different superscripts indicate significant differences (p<0.05).

There was a significant increase in consumption of male layer chicken receiving probiotic or AGP supplementations (Figure-2). Moreover, the feed consumption of the T4 and T5 groups was increased higher than that of the T0 and T1 groups. There was no difference in the feed consumption between the T2 and T3 groups and the T4 and T5 groups during the starter phase of this study (Table-2).

**Figure-3:**
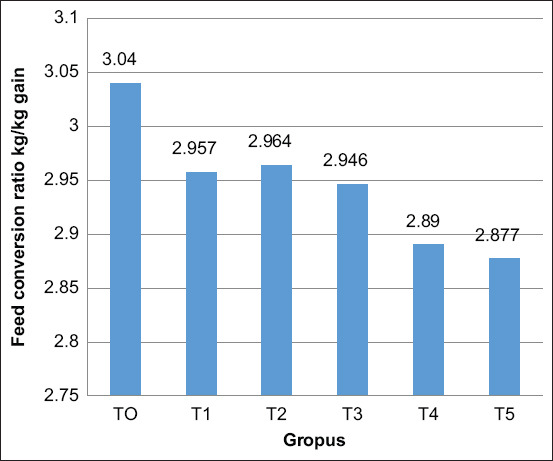
Efficacy of dietary supplementary probiotics on feed conversion ratio of male layer chicken during the starter period. Values are expressed in mean±standard error. One-way analysis of variance was carried out, followed by Duncan’s comparison test. ^a,b,c^Different superscripts indicate significant differences (p<0.05).

Figure-3 shows a decreased FCR of male layer chicken receiving AGP supplementation and even more in those treated with probiotics. The FCR of the T4 and T5 groups was decreased than those of the other treatment groups. These results indicated that probiotic supplementation at 4 or 5 mL/kg feed improved the body weight, FCR, and feed consumption during the starter.

### Carcass traits

In general, probiotic supplementation (*L. acidophilus, L. plantarum*, and *Bifidobacterium* spp.) at 4 and 5 mL/kg feed (T4 and T5) induced an increase in the weight at slaughter of the animal, breast muscles, and liver compared with that measured in the other treatment groups. Furthermore, the heart, kidney, and lung weights in animals treated with AGP or probiotics increased greater than those of the group receiving 100% basal feed (T0) (Table-3).

**Table 3:** Carcass traits, immune organs, and non-edible organs of male layer chicken fed different experimental diets during the starter period.

Variables	T0	T1	T2	T3	T4	T5
Weight at slaughter (g)	294.40±4.73^a^	319.23±3.70^b^	319.37±5.57^b^	319.45±5.28^b^	352.31±2.37^c^	352.73±3.48^c^
Carcass weight (g)	216.89±4.04^a^	225.46±2.43^b^	226.79±2.78^b,c^	226.64±2.35^b,c^	227.87±1.56^c^	228.15±2.29^c^
Dressing percentage	65.71	65.63	67.99	68.27	68.4	68.5
In carcasses (g/100 g body weight)						
Breast muscles	0.645±0.009^a^	0.714±0.007^b^	0.722±0.009^b^	0.728±0.008^b^	0.737±0.005^c^	0.740±0.007^c^
Liver	0.07691±0.0014^a^	0.09207±0.0010^b^	0.09245±0.0011^b,c^	0.09239±0.0010^b,c^	0.09289±0.0006^b,c^	0.09300±0.0009^c^
Heart	0.014523±0.0003^a^	0.018207±0.0002^b^	0.018214±0.0002^b^	0.018203±0.0002^b^	0.018302±0.0001^b^	0.018324±0.0002^b^
Lungs	0.01452±0.0003	0.01752±0.0002	0.01787±0.0002	0.01786±0.0002	0.01796±0.0001	0.01793±0.0002
Kidney	0.02013±0.0004	0.02199±0.0002	0.02200±0.0003	0.02198±0.0002	0.02210±0.0002	0.02213±0.0002
Immune organ (g/100 g body weight)						
Spleen	0.00693±0.00013^a^	0.00721±0.00008^b^	0.00722±0.00009^b^	0.00721±0.00007^b^	0.00725±0.00005^b^	0.00726±0.00007^b^
Thymus	0.01915±0.0004^a^	0.01923±0.0002^b^	0.01994±0.0002^b^	0.01991±0.0002^b^	0.02107±0.0001^c^	0.02213±0.0002^c^
Bursa of Fabricius	0.01452±0.0003^a^	0.01717±0.0002^b^	0.01718±0.0002^b^	0.01720±0.0002^b^	0.01727±0.0001^b^	0.01729±0.0002^b^
Non-edible organ (g/100 g body weight)						
Head	0.1786±0.0033^a^	0.1879±0.0020^b^	0.1880±0.0023^b^	0.1879±0.0019^b^	0.1889±0.0013^b^	0.1891±0.0019^b^
Leg	0.1997±0.0037^a^	0.2092±0.0023^b^	0.2093±0.0026^b^	0.2092±0.0022^b^	0.2103±0.0014^b^	0.2106±0.0021^b^
Wing	0.3070±0.0057^a^	0.3308±0.0036^b^	0.3310±0.0041^b^	0.3307±0.0034^b^	0.3325±0.0023^b^	0.3329±0.0033^b^

^a,b,c^ Different superscripts in the same raw show significant difference (p<0.05). (T0) 100% basal feed, (T1) basal feed+2.5 g AGP/kg feed, (T2) basal feed+probiotic 1 mL/kg feed, (T3) basal feed+probiotics 3 mL/kg feed, (T4) basal feed+probiotic 4 mL/kg feed, and (T5) basal feed+probiotic 5 mL/kg feed. AGP=Antibiotic growth promoter

### Immune organs

There were significant differences between the T0 group and the other treatment groups (p<0.05) (Table-3) regarding the weight of immune organs, including the spleen, bursa of Fabricius, and thymus. The lowest weights were found for the spleen, bursa of Fabricius, and thymus of the T0 group (100% basal feed). In contrast, the spleen and bursa of Fabricius weights of animals receiving probiotic and AGP supplementations (T1, T2, T3, T4, and T5) were the greatest. The thymus weight was also increased in the groups treated with probiotics at 4 and 5 mL/kg feed (T4 and T5) than in other treatment groups (Table-3).

### Non-edible organs

The weight of the head, legs, and wings was more significant in groups receiving probiotic or AGP supplementation (T1, T2, T3, T4, and T5) than those measured in the basal feed group (T0) (Table-3).

## Discussion

In this study, the supplementation of feed with probiotics (*L. acidophilus*, *L. plantarum*, and *Bifidobacterium* spp.) in male layer chicken during the starter period led to an increase in feed consumption and body weight and a decrease of the FCR. These probiotics might reduce the FCR and increase the body weight by improving the digestibility and absorption of nutrients in male layer chicken during the starter period. As shown previously [21], the use of probiotics leads to significant differences in growth performances, weight gain, feed intake, and FCR during the starter phase. The higher feed consumption has been linked to a decreased gastric emptying time when probiotics are provided in the feed [22].

In addition, *Lactobacillus* use was reported to support the host cecal microbiota effects of increasing feed efficiency and body weight and decreasing FCR [23-25]. The administration of *Lactobacillus* spp. and *Bacillus cereus* in broiler chicken also reduces the pH of the ileum and cecum by increasing fatty acids. This has a positive antibacterial impact as it reduces the amount of *Escherichia coli* [26-28]. The growth of pathogenic bacteria is indeed inhibited in an acidic environment in the digestive tract, allowing beneficial bacteria to be dominant in the digestive tract. The decreased pH in the digestive tract also increases the motility of the intestinal wall layer so that the surface area, and consequently the absorption of the intestinal wall increases [29,30]. Therefore, probiotic supplementation positively impacts the development of the intestinal tract in the starter period by maintaining the balance of the gastrointestinal microbiota, which leads to increased feeding and weight gain in broilers [30,31].

The positive impact on body weight and feed conversion might be influenced by the ability of probiotic bacteria to produce enzymes such as cellulases, amylases, and proteases, which can significantly increase nutrient digestibility and increase the body weight of broilers [24,32,33]. However, several studies showed that probiotic supplementation reduces feed consumption [25] and increases FCR in broilers [34]. These different effects of diet enrichment with probiotics might be caused by several factors, including the chicken sex, the chicken developmental phase, the dose of probiotics, and the type of probiotics [24,31].

The weight at slaughter and the carcass, liver, and heart were increased in groups treated with AGP (T1) or probiotics (T2, T3, T4, and T5) compared with those of the control T0 group. The increase in weight at slaughter and carcass might be caused by the increased nutrient digestibility, enzyme activity, and a favorable balance of the gastrointestinal microbiota induced by the addition of probiotics in the feed [3,35]. These data are in agreement with a previous work [36] showing an increase in carcass yield and relative weight of the intestinal tract, as well as a decrease in abdominal fat in the fermented canola meal group, compared with those of the groups receiving canola meal and canola meal with probiotics. The addition of probiotics in feed might lead to a higher carcass weight by increasing the availability of protein in the body [37]. In addition, probiotic inclusion significantly affects growth responses, including body weight and nutrient digestibility [38], and carcass weight [14]. However, the effects of probiotic administration on carcass quality, slaughter weight, breast weight, and organs (liver, heart, and gizzard) were significantly related to the sex of chickens [39-41].

A significantly higher weight of immune organs (bursa of Fabricius) has been shown in animals fed with fermented canola meal and canola meal supplemented with probiotics compared with the weight in other treatment groups and control group [36]. The increase in the relative weight of the bursa of Fabricius is likely due to an increase in the lymphocyte levels induced by the addition of probiotic microbiota to stimulate the immune response of chickens [26,42]. The weight of other immune organs, such as the spleen and thymus, was not significantly different among treatment groups [36]. However, after probiotic administration, weighing lymphoid organs (spleen, bursa of Fabricius, and thymus) showed a significant change in the spleen relative weight on 42 days of age compared to the control group. This might be because the function of lymphoid organs develops with the developmental age of chickens [42]. However, different results were also obtained [43], showing no significant effect on the spleen weight of probiotics addition in chicken feed.

Non-edible body parts of chickens include bones and slaughter offal (feathers, wings, head, and leg) [44]. In the present study, the administration of probiotics affected, albeit small, on the weight of the wings, head, and leg. This result most likely reflects the positive correlation between body weight and linear body measurements, which means that all linear body traits increase simultaneously with the chicken body weight [44]. This agrees with the previous reports [45,46] of a positive and significant correlation between linear body size and body weight. However, there are conflicting findings regarding the positive correlation between linear body parameters and body weight in poultry [47,48]. The differences in body weight indicate that the administration of probiotics to male layer chicken at the starter period affects chickens and consequently affects the measurement of other organs, especially non-edible organs.

## Conclusion

Probiotic (*L. acidophilus*, *L. plantarum*, and *Bifidobacterium* spp.) supplementation during the starter period might improve the growth, carcass traits, and immune organ size of male layer chicken. It is necessary to conduct more in-depth study on the level of digestibility and activity of digestive enzymes with the addition of probiotics in male layer chicken of the ISA Brown strain.

## Authors’ Contributions

BA: Supervised the study. WPL, MNY, AEC, and RNK: Conducted the study. WPL: Statistical analysis of the data. SW: Drafted the manuscript. WPL and MNY: Revised the manuscript. All authors read and approved the final manuscript.
